# Accidental cystectomy during laparoscopic excision of prostatic utricle cyst - a rare complication

**DOI:** 10.1590/S1677-5538.IBJU.2017.0284

**Published:** 2018

**Authors:** Vikash Kumar, Chirag Punatar, Kunal Jadhav, Sharad Sagade

**Affiliations:** 1 Department of Urology, P. D. Hinduja National Hospital and Medical Research Centre, Mumbai, Maharashtra, India

**Keywords:** Cystectomy, Intraoperative Complications, Prostate

## Abstract

Prostatic utricle cyst is a rare congenital anomaly. Symptomatic cysts require treatment. Surgical excision is the treatment of choice, but is challenging due to close proximity to vas deferens, ejaculatory ducts, bladder, prostate, rectum and pelvic nerves. Complications include rectal injury, ureteral injury, impotence, infertility and faecal incontinence. We here report a rare complication in which bladder was accidentally removed during laparoscopic excision of prostatic utricle cyst. To best of our knowledge such a complication has never been reported previously. We also describe the possible cause of this accident and suggest ways to prevent this disastrous complication.

## INTRODUCTION

Prostatic utricle cyst is a rare congenital anomaly. Cysts vary in size and presentations differ. Symptomatic cysts require treatment (1). Surgical excision is treatment of choice. Surgery can have its own complications. We report a rare case of accidental urinary bladder cystectomy during laparoscopic excision of prostatic utricle cyst.

## CASE REPORT

**Figure 1:**
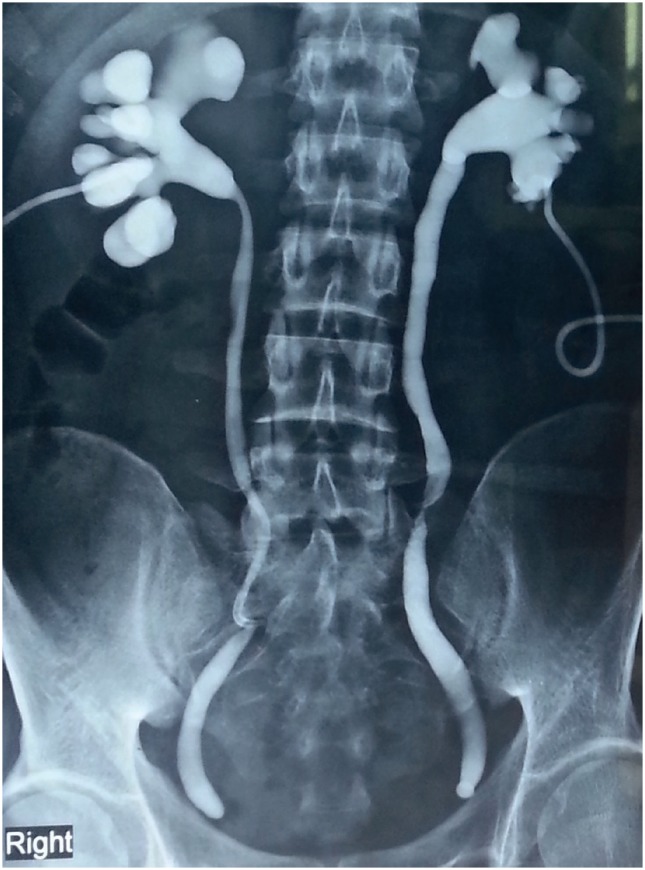
Nephrostomogram showing complete cut off at the level of lower ureter on both sides (done after being operated elsewhere).

A 24 year old male presented to us with urinary diversion by bilateral percutaneous nephrostomies (PCN), performed six months ago. He had undergone laparoscopic surgery for removal of prostatic utricle cyst elsewhere. Postoperatively he developed anuria. A sonogram revealed bilateral hydro-ureteronephrosis. Bladder was not commented upon. This acute crisis was treated by bilateral PCN. Nephrostomogram revealed complete cut-off of both lower ureters (Figure-1).

**Figure 2:**
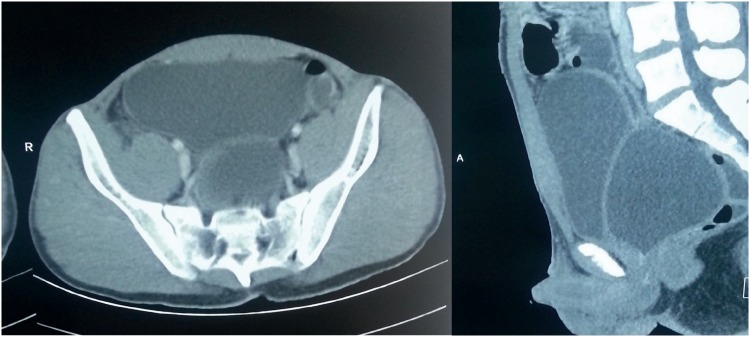
Preoperative CT scan showing the bladder anteriorly and prostatic utricle cyst posteriorly.

He had history of lower abdominal pain with burning micturition on and off since two years. Investigations had revealed a prostatic utricle cyst with infection. Following conservative management, he was asymptomatic for about 18 months. Recurrence of symptoms was associated with increase in cyst size (Figure-2). Surgical treatment was advised at this time. Laparoscopic cyst excision was undertaken which resulted in anuria leading to emergency bilateral PCN. Patient presented to us six months later.

**Figure 3:**
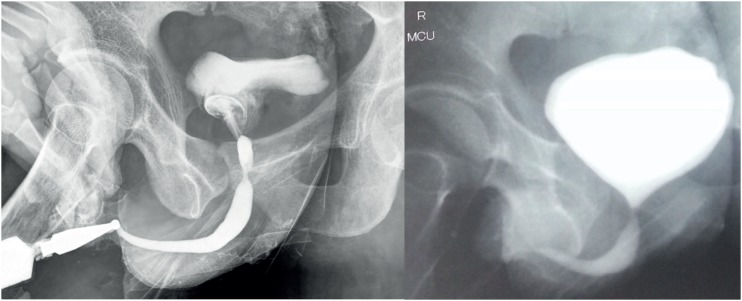
Ascending cysto-urethrogram and MCU showing normal anterior urethra and a smooth walled small capacity bladder (which in retrospect was actually the prostatic utricle cyst).

Systemic examination was normal. Abdominal examination revealed port site scars, bilateral nephrostomies and coronal hypospadias. Investigations revealed normal hemogram and creatinine. Bilateral lower ureteric injury was the suspected diagnosis initially. Ascending and micturating cysto-urethrogram (MCU) showed smooth walled bladder with mildly reduced capacity and normal urethra (Figure-3).

With evidence of bilateral ureteric cut-off and normal lower urinary tract, bilateral ureteric re-implantation was planned. Urethrocystoscopy showed normal anterior urethra. There was an opening on verumontanum, which accommodated 17 French cystoscope sheath easily. This lead to a smooth walled cavity containing about 200 mL of turbid fluid. The epithelium was not like normal urothelium. Ureteric orifices were not seen. Then we realized that this cavity was indeed the cyst which was falsely mistaken as bladder on MCU. The proximal urethra was completely cut off below the level of bladder neck, ending blindly. A situation of accidental urinary bladder cystectomy and not prostatic utricular cystectomy was realized. Further surgery was abandoned.

**Figure 4:**
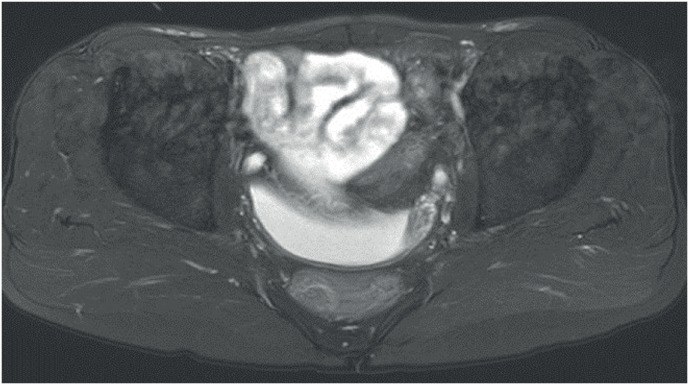
MRI showing the ureters anteriorly and the prostatic utricle cyst posteriorly. The bladder is absent.

Patient was explained about absence of urinary bladder. MRI pelvis confirmed the same (Figure-4). Surgical options were discussed and he opted for orthotopic neo-bladder. Ureters were dissected, prostatic utricle cyst was marsupialized, it's opening into urethra closed and Studer's orthotopic ileal neobladder was constructed.

**Figure 5:**
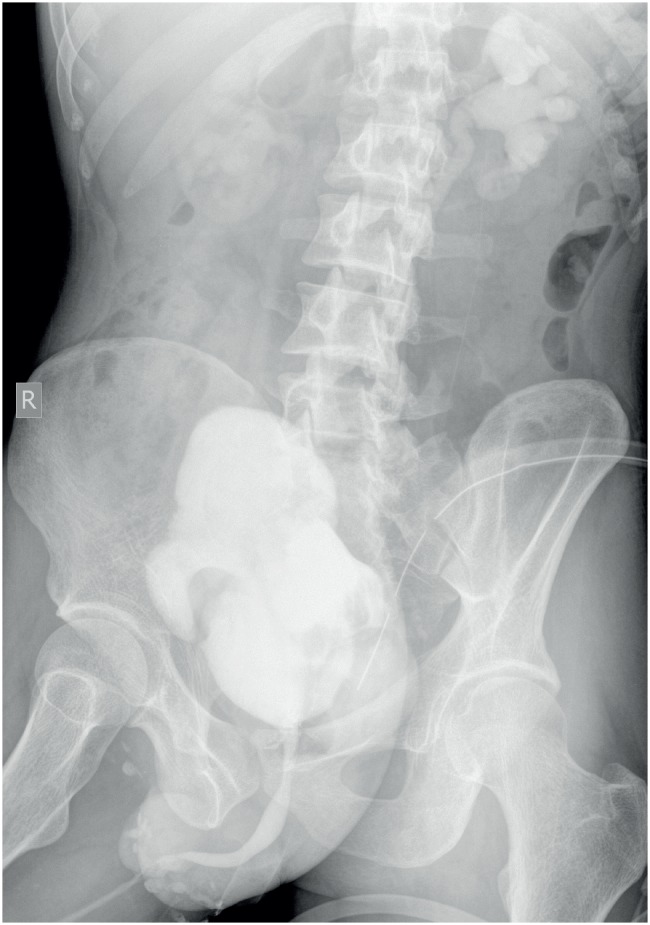
Postoperative MCU showing a good capacity neobladder with no extravasation. The urethra appears normal. There is reflux of contrast on both sides.

Postoperative MCU showed good capacity neobladder and no extravasation (Figure-5). Nephrostomies were clamped and removed.

At follow-up, he was voiding well with minimal residue and no incontinence. His outcomes in terms of ejaculation are yet not known.

## DISCUSSION

Prostatic utricles are remnants of Mullerian ducts. Normally Mullerian ducts regress under influence of mullerian regression factor and is represented by appendix testis (cephalad part) and utricle (caudal part). Utricular anomalies result from incomplete regression of Mullerian ducts or incomplete androgen mediated closure of the urogenital sinus in form of prostatic utricular cyst (2).

Mullerian duct remnants are uncommon. Incidence of enlarged prostatic utricle is 11-14% in association with hypospadias or intersex anomalies, more so with perineal hypospadias (>50%). About 10-25% show an association with renal agenesis / dysgenesis and 25% cases with hypospadias (1).

Cysts are quite often asymptomatic. They may present with lower urinary tract symptoms, urinary retention, stone formation, epididymitis, obstructive azoospermia, rectal mass, and rarely malignant growth. Symptoms are determined by degree of obstruction of bladder neck or seminal vesicles and ejaculatory ducts, and infection (2). The relation of cyst size and symptoms is debated (1, 2).

Symptomatic cysts require intervention. Modalities include endoscopic deroofing, transrectal ultrasound guided or transperineal cyst aspiration and sclerotherapy, endoscopic fulguration of cyst lining (1, 2), marsupialisation of cyst into bladder (3), open / laparoscopic (4) excision of cyst, and recently robot assisted surgical excision (5).

Surgical excision is the treatment of choice, but is challenging due to close proximity to vas deferens, ejaculatory ducts, bladder, prostate, rectum and pelvic nerves. Approaches described include abdominal extravesical, transvesical (transtrigonal), perineal and anterior or posterior transrectal sagittal approaches. Complications include rectal injury, ureteral injury, impotence, infertility and faecal incontinence (2).

In our patient there was accidental removal of urinary bladder. To best of our knowledge, such complication has never been reported. Possibly Foley catheter was lodged into utricular cyst, misleading the surgeon to excise urinary bladder.

Preoperative cystourethroscopy could have shown that opening of utricle cyst was directly in line with urethra, bladder neck was high at an angle, and would have more clearly shown anatomical relationship of cyst with bladder.

Preoperative cannulation of cyst has been described to facilitate laparoscopic identification and mobilization of cyst (4).

Intraoperative flexible cystoscopy helps in identifying the bladder from cyst, by seeing light of cystoscope during laparoscopy. By chance, if bladder gets injured, it could be identified immediately.

Preoperative cannulation of ureters with ureteral catheters helps in identifying them intraoperatively. By seeing the structure into which ureters enter, bladder could have been differentiated from cyst. Ureteral injury could be identified intraoperatively.

Adopting above mentioned measures could potentially avoid such disastrous complication.
